# Draft Genome Sequence of Taylorella equigenitalis Strain 210217RC10635, Isolated from a Pony Stallion in Germany

**DOI:** 10.1128/MRA.01112-18

**Published:** 2018-09-27

**Authors:** Falk Melzer, Astrid Raßbach, Astrid Köenig-Mozes, Mandy Carolina Elschner, Herbert Tomaso, Anne Busch

**Affiliations:** a Friedrich-Loeffler-Institut, Institute of Bacterial Infections and Zoonoses (IBIZ), Jena, Germany; b Landesuntersuchungsamt Rheinland-Pfalz, Koblenz, Germany; Indiana University Bloomington

## Abstract

Here, we report the draft genome sequence of Taylorella equigenitalis strain 210217RC10635, a Gram-negative bacterium belonging to the genus Taylorella and the order Burkholderiales. Taylorella equigenitalis is the causative agent of contagious equine metritis (CEM). The strain reported here was isolated in 2017 from a German stallion.

## Abstract (precis)

Here, we report the draft genome sequence of Taylorella equigenitalis strain 210217RC10635, a Gram-negative bacterium belonging to the genus Taylorella and the order Burkholderiales. Taylorella equigenitalis is the causative agent of contagious equine metritis (CEM).

## ANNOUNCEMENT

Taylorella equigenitalis is classified in the Burkholderiales order and the Alcaligenaceae family. It is the etiological agent of contagious equine metritis (CEM), a highly contagious, sexually transmitted infection of horses. The infected mares show clinical signs of mucopurulent vaginal discharge and various degrees of vaginitis, cervicitis, and endometritis, and it may result in temporary infertility or early embryonic death (1, 2). In stallions, no clinical signs are reported because the stallions are not strictly infected, as T. equigenitalis merely colonizes predilection sites in the external genitalia without eliciting an immune response. CEM is a disease notifiable to the World Organisation for Animal Health (OIE) and is considered part of veterinary certification for international trade purposes. We report here the genome sequence of Taylorella equigenitalis strain 210217RC10635, which was isolated in 2017 from a pony stallion from Rhineland-Palatinate, Germany. It was isolated from a swab sample of the fossa glandis. Sequence typing of whole-genome sequences of strain 210217RC10635 using the Taylorella MLST databases (https://pubmlst.org/taylorella) (3) revealed its membership in a previously unknown sequence type with a 100% identity (Table 1).

**TABLE 1:** Sequence typing data for Taylorella equigenitalis strain 210217RC10635^*a*^

Locus	HSP length (bp)^*b*^	Allele length (bp)	Allele
*adk*	538	538	*adk_2*
*fh*	574	574	*fh_4*
*glta*	623	623	*glta_1*
*gyrb*	518	518	*gyrb_3*
*shmt*	435	435	*shmt_1*
*txn*	371	371	*txn_4*
*tyrb*	462	462	*tyrb_2*

a All loci are covered with 100% identity and no gaps.

b HSP, high-scoring segment pair.

DNA for whole-genome sequencing was prepared from colonies grown on a chocolate blood agar culture plate using a High Pure PCR template preparation kit (Roche Diagnostics GmbH, Mannheim, Germany). The sequencing library was generated using the Nextera XT DNA library prep kit (Illumina, Inc., San Diego, CA, USA). From an Illumina MiSeq run with an average read length of 300 bp and an expected insert size of 350 bp, 1.3 million paired-end reads were generated (mean sequencing depth of >180 reads with a standard deviation of 60 bp). Further processing included quality trimming and assembly (included in SPAdes version 3.9.1 in Bayes Hammer mode [4]) and filtering of the samples to remove contigs with coverage less than 5 and a size below 500 bases. The genome assembly was represented by 13 contigs, with an *N*_50_ contig length of 400,994 bp, and the largest contig was 451,743 bp. Annotation was performed with Prokka (5), and annotation features include 1,475 genes and 3 rRNAs, 34 tRNAs, and 1 transfer-messenger RNA (tmRNA) for a total sequence length of 1,619,593 bp. The GC content of the draft genome sequences is 37.51%.

### Data availability.

Taylorella equigenitalis 210217RC10635 was deposited at BioProject under the accession number PRJNA477933, at BioSample under the accession number SAMN09487631, and at GenBank under the accession number QMFX00000000. Primary data were deposited at the NCBI primary data archive SRA, with the reference run number SRP158361. The version described in this paper is the first version.
